# Estrogen and progesterone receptors have distinct roles in the establishment of the hyperplastic phenotype in PR-A transgenic mice

**DOI:** 10.1186/bcr2408

**Published:** 2009-09-29

**Authors:** Marina Simian, Mina J Bissell, Mary Helen Barcellos-Hoff, Gopalan Shyamala

**Affiliations:** 1 Life Sciences Division, Lawrence Berkeley National Laboratory, 1 Cyclotron Road, Berkeley, CA 94720, USA; 2 Current address: Research Area, Instituto de Oncología 'Ángel H. Roffo', Avda San Martín 5481, Buenos Aires C1417DTB, Argentina; 3 Current address: Department of Radiation Oncology, New York University Langone Medical Center, 566 First Avenue, New York, NY 10016, USA

## Abstract

**Introduction:**

Expression of the A and B forms of progesterone receptor (PR) in an appropriate ratio is critical for mammary development. Mammary glands of PR-A transgenic mice, carrying an additional A form of PR as a transgene, exhibit morphological features associated with the development of mammary tumors. Our objective was to determine the roles of estrogen (E) and progesterone (P) in the genesis of mammary hyperplasias/preneoplasias in PR-A transgenics.

**Methods:**

We subjected PR-A mice to hormonal treatments and analyzed mammary glands for the presence of hyperplasias and used BrdU incorporation to measure proliferation. Quantitative image analysis was carried out to compare levels of latency-associated peptide and transforming growth factor beta 1 (TGFβ1) between PR-A and PR-B transgenics. Basement membrane disruption was examined by immunofluorescence and proteolytic activity by zymography.

**Results:**

The hyperplastic phenotype of PR-A transgenics is inhibited by ovariectomy, and is reversed by treatment with E + P. Studies using the antiestrogen ICI 182,780 or antiprogestins RU486 or ZK 98,299 show that the increase in proliferation requires signaling through E/estrogen receptor alpha but is not sufficient to give rise to hyperplasias, whereas signaling through P/PR has little impact on proliferation but is essential for the manifestation of hyperplasias. Increased proliferation is correlated with decreased TGFβ1 activation in the PR-A transgenics. Analysis of basement membrane integrity showed loss of laminin-5, collagen III and collagen IV in mammary glands of PR-A mice, which is restored by ovariectomy. Examination of matrix metalloproteases (MMPs) showed that total levels of MMP-2 correlate with the steady-state levels of PR, and that areas of laminin-5 loss coincide with those of activation of MMP-2 in PR-A transgenics. Activation of MMP-2 is dependent on treatment with E and P in ovariectomized wild-type mice, but is achieved only by treatment with P in PR-A mice.

**Conclusions:**

These data establish a link between hormonal response, proliferation, modulation of MMP activity and maintenance of basement membrane integrity that depend on a balance in the expression levels of PR-A and PR-B isoforms. Notably, concomitant increased proliferation, due to inhibition of TGFβ1 activation, and loss of basement membrane integrity, via increased MMP-2 activity, appear to be prerequisites for the PR-A hyperplastic phenotype.

## Introduction

Progesterone receptor (PR) belongs to the superfamily of steroid receptors and mediates the action of progesterone in its target tissues [1,2]. In both humans and rodents, progesterone promotes the proliferation of epithelial cells that accompanies each menstrual/estrous cycle and pregnancy. In normal mammary glands of adult human and rodent females, PR expression is restricted to the luminal epithelial cells of the duct [3]. Studies on PR-null mutant mice have revealed that PR is essential for progesterone-dependent proliferation of epithelial cells [4].

PR exists in two isoforms, the A and B forms, and the expression of these, in an appropriate ratio, is critical for normal mammary development [5]. As such, mammary development is abnormal in transgenic mice carrying either an additional A form of PR (PR-A transgenics) or the B form of PR (PR-B transgenics) [6,7]. In particular, mammary glands of PR-A transgenics are characterized by extensive lateral branching, ductal hyperplasia, a disorganized basement membrane (BM) and loss of cell-cell adhesion [6]. Studies using the molecular markers for transformation, as defined by Medina [8], revealed that these mammary glands contained at least two distinct populations of transformed epithelial cells. The ducts with normal histology contain cells resembling immortalized cells, while hyperplasias consist of cells in later stages of transformation associated with early preneoplasias [9].

The development of cancer is also associated with disruption of tissue architecture. Branching morphogenesis in the mammary gland is the culmination of hormone-mediated proliferation and extracellular matrix (ECM) remodeling; these are each in turn dependent on the production of growth factors and the balance between ECM production and degradation [10]. Once established, the mammary gland undergoes rounds of highly orchestrated proliferation and morphogenesis during pregnancy and involution, yet without losing the fundamental patterning of the gland. In contrast, hyperplasia is defined as loss of this patterning and is considered to be a precursor to neoplasia.

It is well established that PR-A can modulate the activities of both estrogen receptor (ER) alpha and PR [11,12]. Accordingly, either estrogen action or progesterone action or both, resulting from overexpression of PR-A, may mediate the abnormal mammary phenotype of PR-A transgenics. To this end, the objective of our present study was to identify the respective roles of estrogen and progesterone in the genesis of mammary hyperplasias/preneoplasias in PR-A transgenic mice.

## Materials and methods

### Mice, treatment with steroids and tissue preparations

All mice used in these studies were of FVB strain. The mice were housed and cared for in accordance with the National Institutes of Health guide to humane use of animals in research. All experiments were conducted with Lawrence Berkeley National Laboratory institutional review and approval. Animals were killed by carbon dioxide inhalation and cervical dislocation at the indicated times in accordance with Association for Assessment and Accreditation of Laboratory Animal Care guidelines.

PR-A transgenic mice [6] and PR null mutant mice [4] have been described previously. Nulliparous adult (9 to 12 weeks old) mice were used either intact or ovariectomized and/or treated with steroids for the indicated times. For zymography studies, prior to tissue collection, mice were perfused with 10 ml PBS and the tissues were frozen in liquid nitrogen and stored at -70°C until use.

For studies with steroids, nulliparous adult mice were used either as intact or after ovariectomy. Estradiol (1 μg/mouse) and/or progesterone (1 mg/mouse) were administered as described previously [3]. Antiestrogen, ICI 182,780 (50 μg/mouse; Tocris Cookson Inc., Ellisville, MO, USA) or antiprogestin, mifepristone (RU486, 16 μg/g body weight; Sigma, St Louis, MO, USA) or ZK 98,299 (16 μg/g body weight; gift from Dr Ming-Wei Wang) were administrated daily for 4 days. For cell proliferation studies, mice were administered 160 μg/g body weight 5-bromo-2-deoxyuridine (BrdU) (Sigma) 2 hours prior to sacrifice.

For mammary whole mounts, one of the Number 4 inguinal mammary glands was fixed in Carnoy's solution and stained in Alum Carmine [0.2% carmine/0.5% aluminum potassium sulfate (both from Sigma, St Louis, MO, USA)]. For immunohistochemical analyses on paraffin sections, mammary tissues were collected, fixed in 4.7% buffered formalin (Fisher Scientific, Pittsburgh, PA, USA), dehydrated, embedded in paraffin and cut into 5 μm thick sections. For immunofluorescence analyses on frozen sections, the entire Number 4 inguinal mammary glands were mounted and quick-frozen in Optimal Cutting Temperature compound (Ted Pella, Inc., Redding, CA, USA). Cryostat sections (5 to 10 μm thick) were cut and mounted onto glass slides and were fixed for 2 minutes in methanol/acetone (1:1).

### Antibodies

The antibodies used were: anti-BrdU, rat monoclonal antibody (Harlan Sera-Lab Ltd, Loughborough, UK); goat anti-latency-associated peptide (anti-LAP) and chicken anti-transforming growth factor beta 1 (anti-TGFβ1) (R&D Systems, Minneapolis, MN, USA); MMP-2 (Millipore, Bilerica, MA, USA); collagen I, collagen III and collagen IV (Southern Biotechnology Assoc. Inc., Birmingham, AL, USA); laminin-1 (Telios Pharmaceuticals, San Diego, CA, USA); and laminin-5 (gift from Dr V Quaranta).

### Immunohistochemistry and immunofluorescence

BrdU-positive cells were analyzed in paraffin-embedded sections as described previously [9]. Briefly, paraffin sections were deparaffinized and rehydrated prior to antigen retrieval and treatment with specified antibodies. The antigen-antibody complexes were identified using the Universal DAKO LSAB2-labeled streptavidin-biotin peroxidase kit (DAKO, Carpinteria, CA, USA). The sections were counterstained with Mayer's hematoxylin solution (DAKO). After counterstaining, nuclei negative for the antigen appeared purple-blue and positive nuclei appeared brown.

Immunofluorescence assays were performed as described previously [3]. Briefly, nonspecific binding sites were blocked by incubation in a blocking buffer (PBS containing either 1% casein or 15% FCS and 0.2% Tween 20), for 30 minutes at ambient temperature. Sections were then treated with primary antibodies, washed with PBS and incubated with fluorescein isothiocyanate-conjugated secondary antibodies (dissolved in the blocking buffer) for 30 minutes at ambient temperature. Slides were then washed five times with PBS and the nuclei were stained with 4',6-diamino-2-phenylindole and mounted with Vectashield (Vector Laboratories, Burlingame, CA, USA).

### Image acquisition and processing

Immunofluorescence images were obtained using a 40 × 0.75 numerical aperture Zeiss Neofluor objective on a Zeiss Axiovert equipped with epifluorescence (Carl Zeiss MicroImaging, Inc., Thornwood, NY, USA). A multiband-pass dichroic mirror, barrier filter and differential wavelength filter wheel combination was used to selectively excite fluorochromes in sequence. Images were captured using a scientific-grade 12-bit charged coupled device (KAF-1400, 1,317 × 1,035, 6.8 μm square pixels) on a Xillix digital camera (Vancouver, Canada). Relative intensity of images was maintained when constructing figures using Scilimage (TNO Institute of Applied Physics, Delft, the Netherlands) to scale the 12-bit data to a common 8-bit scale using the dataset minimum and maximum. False color was assigned accordingly. Internal standardization was achieved by comparing only images stained with the same antibodies in the same experiment, captured with identical parameters and scaled and displayed identically.

### Zymography

Mammary glands were homogenized on ice in RIPA insoluble buffer (50 mM Tris, pH 8.0, containing 150 mM NaCl, 0.1% sodium dodecyl sulfate, 0.5% deoxycholate and 1% NP40). The homogenates were centrifuged for 15 minutes at 4°C and the supernatants were subjected to electrophoresis on gelatin substrate gels (8.8% sodium dodecyl sulfate-polyacrylamide slab gels containing 1 mg/ml gelatin), as described previously [13]. Subsequently, the gels were treated with 2.5% Triton X-100 for 30 minutes, followed by incubation for 24 hours at 37°C in a buffer containing 100 mM Tris- HCl, pH 7.4, and 15 mM CaCl_2_. The gels were stained with Coomassie Blue R-250 and destained with water until clear zones indicative of proteolytic activity emerged against a blue background. The zymograms were scanned and subjected to densitometric analyses using the PC version of NIH image (Scion Corporation, Frederick, MD, USA).

### Statistical analysis

The data are presented as the mean ± standard error of the mean. The differences between the various experimental groups were analyzed by Student's *t *test and were considered significant when *P *< 0.05 was obtained. A Kolmogorov-Smirnov comparison of two datasets was used to compare the TGFβ1 and LAP data, with *P *< 0.05 considered significant.

## Results

### Mammary phenotype of PR-A transgenics is dependent on ovarian steroids

We previously showed that the mammary glands of adult PR-A transgenic mice are characterized by extensive lateral branching and the presence of very thick ducts [6]. Histological analyses revealed that the thickening of the duct walls was due to the presence of multiple layers of cells, in contrast to the monolayer or simple epithelium found in ducts of wild-type mice [6]. These hyperplasias were characterized by a disruption of the basement membrane and loss of cell-cell adhesion [6].

**Figure 1:**
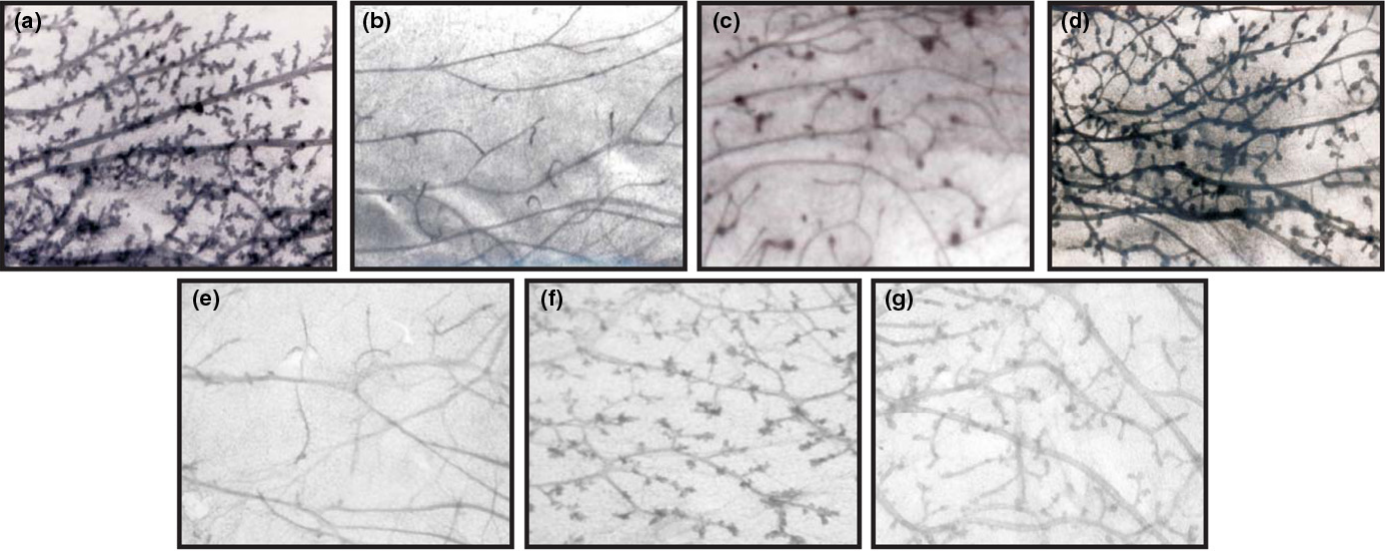
Whole mounts of mammary glands of adult mice carrying additional A form of PR.  Whole mounts of mammary glands derived from **(a) **intact PR-A transgenic mice, **(b) **ovariectomized PR-A transgenic mice, **(c) **ovariectomized PR-A transgenic mice treated with estradiol 1 μg for 5 days, **(d) **ovariectomized PR-A transgenic mice treated with estradiol 1 μg and progesterone 1 mg for 5 days, **(e) **intact PR-A transgenic mice treated with ICI 182,780 for 4 days, **(f) **intact PR-A transgenic mice treated with ZK98,299 for 4 days, and **(g) **intact PR-A transgenic mice treated with RU486 for 4 days.

The morphology of the mammary glands of PR-A transgenics under different hormonal conditions is shown in Figure 1. The excessive ductal side branching in glands of adult females (Figure 1a) was abolished upon ovariectomy (Figure 1b). Short-term administration of estradiol alone initiated epithelial proliferation, characterized by the presence of numerous side buds (Figure 1c); however, when progesterone was administered with estradiol, the effects of ovariectomy on the PR-A phenotype was fully reversed such that the hyperplastic mammary phenotype resembled that of intact mice (Figure 1, compare a and d).

To identify the relative importance of signaling through estrogen versus progesterone in eliciting the excessive ductal growth, intact mice were treated with either antiestrogen ICI 182,780 or antiprogestins RU486 or ZK 98,299. Surprisingly, in mammary glands of mice treated with ICI 182,780, the ductal growth was greatly diminished and was comparable with that of ovariectomy (Figure 1, compare b and e). However, mammary glands of mice treated with ZK 98,299 (Figure 1f) or with RU486 (Figure 1g) still exhibited side branching similar to intact untreated mice.

**Figure 2:**
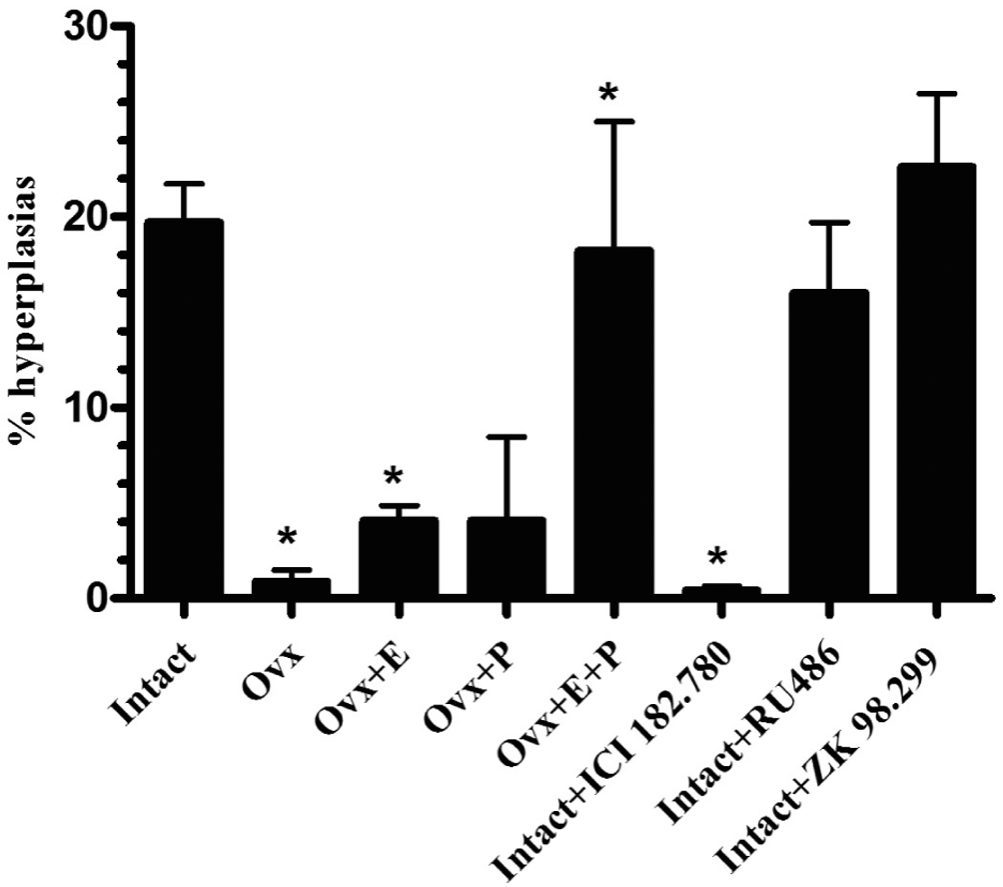
Effects of hormonal treatments on hyperplasia prevalence in mammary glands of PR-A transgenic mice.  Five to six tissue sections, corresponding to each group and representing mammary glands from two to three PR-A transgenic mice, were stained with H&E and a minimum of 100 ducts per section were counted. Each mammary gland from each mouse was analyzed in duplicate. Data is presented as percentage of total ducts and represent the mean ± standard error of the mean. Ovariectomy led to a decrease in the percentage of hyperplasias (intact vs. ovariectomized (Ovx), *P ≤ 0.01), which was partially recovered by administration of estradiol (Ovx vs. Ovx+E, *P ≤ 0.01) and was completely recovered by treatment with estradiol 1 μg and progesterone 1 mg (Ovx vs. OvxE+P, *P ≤ 0.001). On the other hand, treatment with ICI 182,780 also led to a decrease in the percentage of hyperplasias (intact vs. intact+ICI 182,780, *P ≤ 0.001), but neither the antiprogestins RU486 or ZK 98,299 had an effect. Ovx+P, ovariectomized mouse treated with progesterone 1 mg.

Quantitation of the gross histological appearance confirmed the absence of hyperplasias in mammary glands of ovariectomized PR-A transgenics or intact mice treated with ICI 182,780 and the prevalence of hyperplasias in mammary glands of intact mice treated with RU486 or ZK 98,299, which was similar to untreated intact mice (Figure 2). Likewise, treatment of ovariectomized mice with estradiol alone, but not with progesterone, led to a modest increase in the number of hyperplasias; upon treatment with both estradiol and progesterone, however, hyperplasia prevalence was equivalent to that seen in intact mice. These data suggest that estrogen and progesterone have functionally distinct roles in hyperplasia.

**Figure 3:**
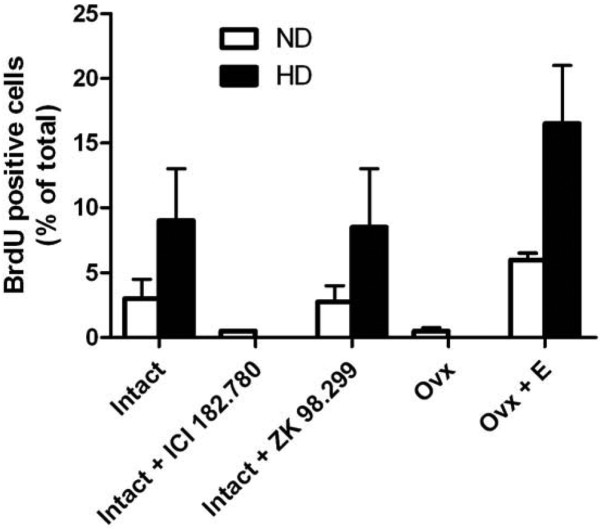
5-Bromo-2-deoxyuridine labeling indices in mammary glands of PR-A transgenic mice under various hormonal statuses.  Percentage of 5-bromo-2-deoxyuridine (BrdU)-positive mammary epithelial cells of normal ducts (ND) and hyperplastic ducts (HD) of PR-A transgenics under various hormonal statuses. Data presented as the mean ± standard error of the mean. Mammary glands from a minimum of three mice were examined, and mammary glands for each mouse were analyzed in triplicate. In each experiment, the percentage of immunopositive cells was obtained by counting a minimum of 500 cells per gland. Ovx, ovariectomized mouse; Ovx+E, ovariectomized mouse treated with estradiol 1 μg.

In parallel analyses for cell proliferation, BrdU-positive cells were rarely detected in mammary glands of ovariectomized mice, but increased upon treatment with estradiol into the range seen in intact mice (Figure 3). Similar to ovariectomy, BrdU-positive cells were infrequent in intact mice treated with the antiestrogen ICI 182,780. The number of BrdU-positive cells in both normal and hyperplastic ducts of intact mice treated with ZK 98,299, however, was essentially similar to that of untreated intact mice (Figure 3). Our earlier studies have shown that antiprogestin, RU486, had no effect on cell proliferation in the hyperplasias [9].

These observations established that a) the increase in cell proliferation, a pre-requisite for the genesis of hyperplasias, required signaling through estradiol/ER but was not sufficient to give rise to overt hyperplasias in magnitude corresponding to that seen in intact untreated PR-A mice; b) that while the overexpression of PR-A leads to the development of the hyperplasias, cross-talk with ER is essential for the manifestation of the hyperplastic phenotype; and c) that antiprogestins ZK 98,299 or RU486 were not sufficient to revert the hyperplastic phenotype. On the whole, these data reveal that, in the context of PR-A overexpression in the mouse mammary gland, the requirement for estradiol and progesterone is mediated through different mechanisms; however, these mechanisms are interdependent.

### TGFβ1 expression is impeded in mammary glands of PR-A transgenic mice

In adult female mice, mammary epithelial cells are maintained in a quiescent state in part due to the actions of TGFβ1 [14]. TGFβ1 is produced as a latent complex with LAP and needs to be activated to exert its biological effect [15]. In the mammary gland, activation of TGFβ1 is regulated by estradiol and progesterone, and restricts the proliferative response to these steroids [14]. The hyperproliferation of mammary epithelial cells in PR-A transgenics is therefore possibly due to diminished expression and/or activity of TGFβ1. One of a few tools available to assess TGFβ1 *in situ *is the double staining for LAP and TGFβ1 epitopes that are revealed upon activation [16].

**Figure 4:**
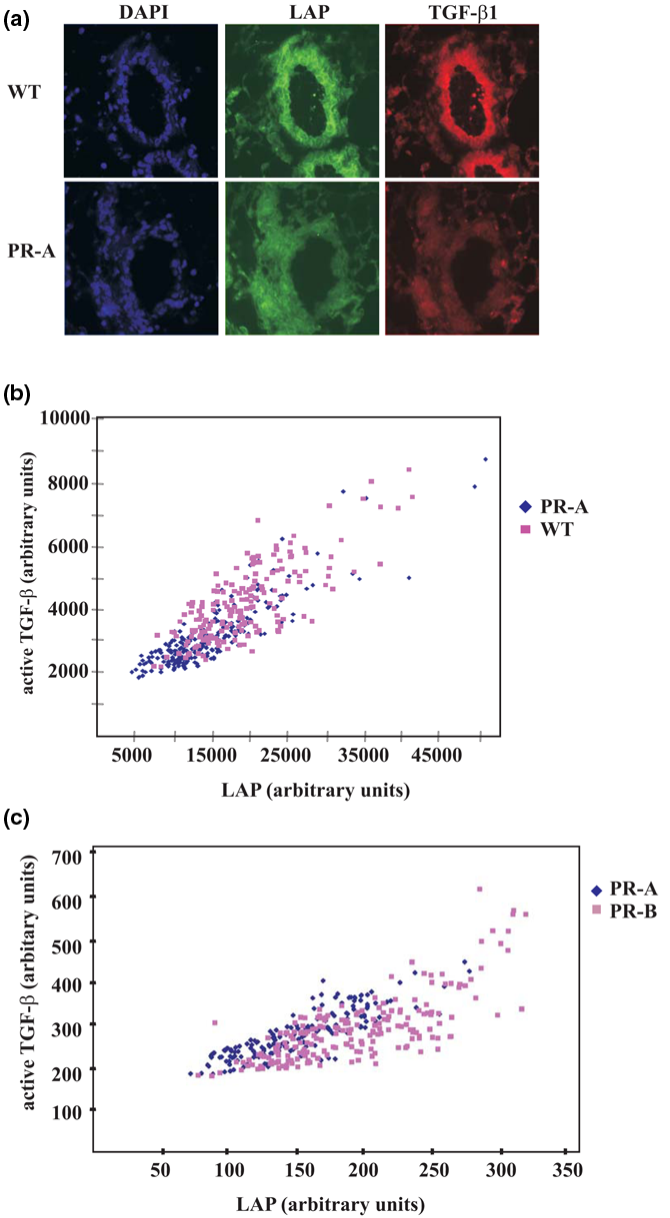
Transforming growth factor beta 1 and latency-associated peptide in PR-A and PR-B mice mammary glands.  **(a) **Examples of dual immunofluorescence staining to latency-associated peptide (LAP) (green) and transforming growth factor beta 1 (TGFβ1) (red) in mammary glands derived from wild-type (WT) and PR-A transgenic mice; both were decreased in the transgenic mice. Nuclei were counterstained with 4',6-diamino-2-phenylindole (DAPI). **(b) **Quantitative image analysis of the intensity of LAP and TGFβ1 immunoreactivity per cell in mammary glands of PR-A mice. Both LAP and TGFβ1 had a lower intensity in cells derived from PR-A transgenic mice as compared with WT controls (*P *< 0.001 in both cases). **(c) **Quantitative image analysis of the intensity of LAP and TGFβ1 immunoreactivity per cell in PR-A mice versus PR-B mice. Only the increase in LAP in PR-B mice compared with PR-A mice was statistically significant (*P *< 0.001). The difference in active TGFβ1, although increased in PR-B mice, was not statistically significant.

Mammary glands of PR-A transgenics showed a statistically significant decrease in both LAP and TGFβ1 as compared with wild-type litter mate controls (Figure 4a, b). Mammary development in PR-B transgenics is abnormal in that they have a limited capacity for ductal elongation, in contrast to hyperplasias exhibited by PR-A transgenics [6,7]. To determine whether reduced expression of TGFβ1 associated with excessive ductal growth in PR-A transgenics was specifically due to overexpression of PR-A, we examined the expression patterns of TGFβ1 in mammary glands of PR-B transgenics. Compared with mammary glands of PR-A transgenics, both latent TGFβ1 and active TGFβ1 were present at a higher level in PR-B transgenics, although only the increase in LAP was statistically significant (Figure 4c). Downregulation of total TGFβ1 mediated by PR-A, in contrast to upregulation by PR-B, therefore probably mimics the *Tgfb1 *heterozygote phenotype of exaggerated response to steroid hormone-mediated proliferation [17].

### Modulation of matrix metalloproteinase activity and basement membrane integrity by estrogen and progesterone

**Figure 5:**
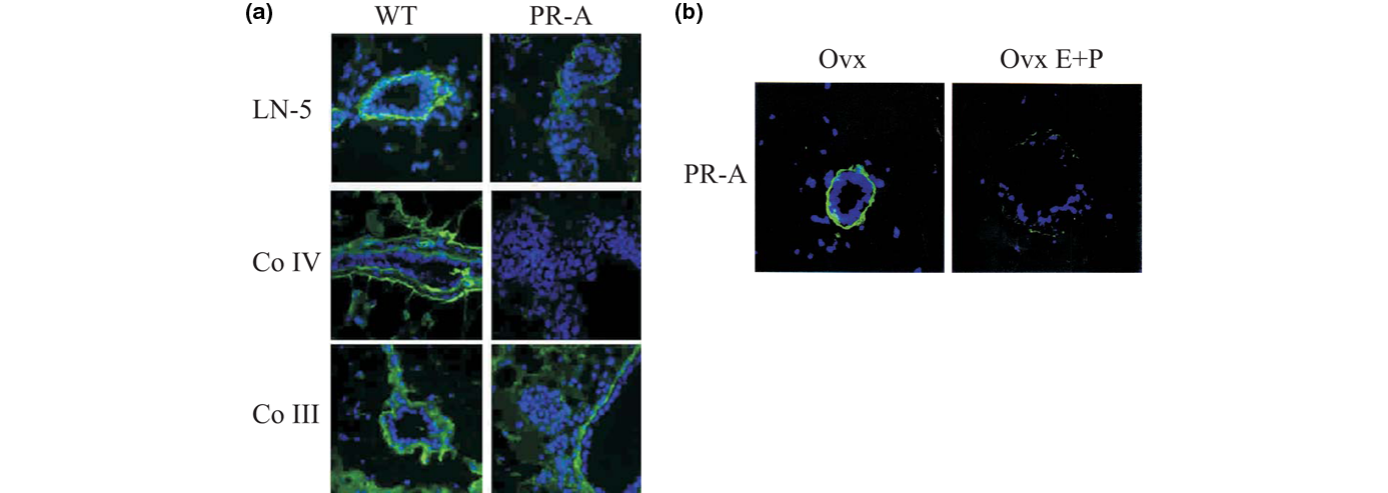
Basement membrane disruption in mammary glands of PR-A transgenic mice: regulation by ovarian hormones.  **(a) **Laminin-5 (LN-5), collagen IV (CoIV) and collagen III (CoIII) immunoreactivity (green) circumscribed the mammary epithelium of wild-type (WT) mice, but were decreased in the glands of PR-A transgenic mice. Nuclei were counterstained with 4',6-diamino-2-phenylindole (blue). **(b) **Ovariectomy (Ovx) of PR-A mice led to a recovery of the basement membrane integrity as shown by the continuous laminin staining. Upon treatment with estrogen 1 μg and progesterone 1 mg for 4 days (OvxE+P), however, the staining was lost.

Side branching and alveolar morphogenesis requires remodeling of the BM [18]. We had previously shown discontinuous laminin-1 staining in the mammary glands of PR-A transgenics indicating a loss of BM integrity [6]. We show here that immunostaining for laminin-5 and collagen IV was also discontinuous. Furthermore, there was decreased collagen III immunoreactivity in the stroma surrounding the regions with aberrant epithelial structures (Figure 5a). Importantly, ovariectomy restored the integrity of the BM as revealed by continuous laminin-1 staining in PR-A mice (Figure 5b), and this effect was reversed by administration of estrogen and progesterone (Figure 5b). Upregulation of PR-A therefore appears to lead to remodeling of the BM.

**Figure 6:**
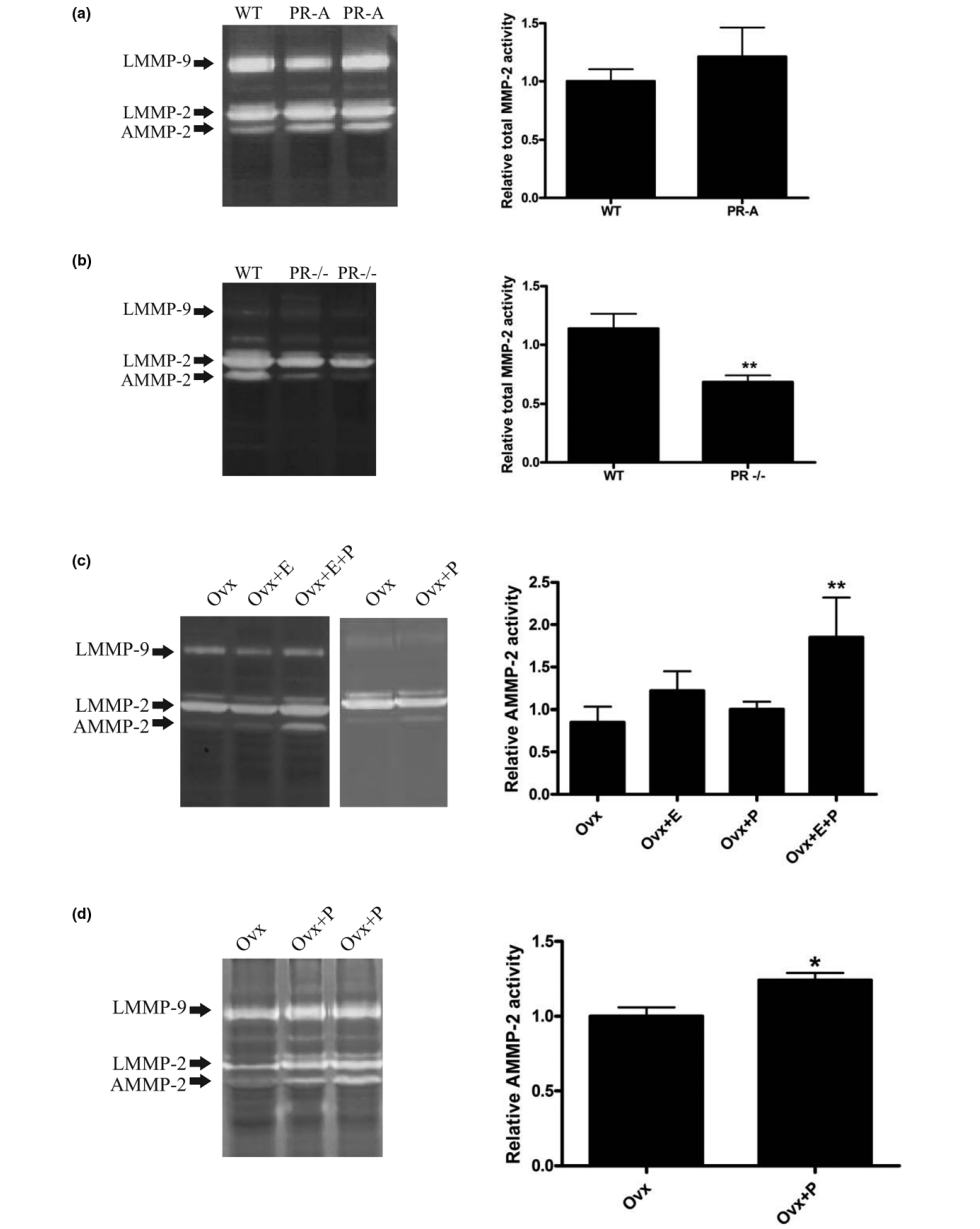
Matrix metalloproteinases in mammary glands of adult wild-type and PR-A transgenic mice analyzed by zymography.  **(a) **Zymogram of protein extracts prepared from mammary glands of two PR-A mice, and a littermate control (WT). Graph shows comparison of the levels, estimated by laser densitometry, of total MMP-2 in transgenic mice (n = 5) and littermate control mice (n = 3). No statistically significant differences were found. Similar results were obtained for MMP-9 (data not shown). **(b) **Zymogram of protein extracts prepared from mammary glands of two PR null mutant mice (PR-^/-^), and a littermate control (WT). Graph shows comparison of the levels, estimated by laser densitometry, of total MMP-2 in PR-^/- ^mice (n = 5) and littermate control mice (n = 4). There was a statistically significant reduction in the total levels of MMP-2 in the PR null mutant mice (*P *≤ 0.01). **(c) **Representative zymograms showing activation of MMP-2 in wild-type mice only when they were treated with estrogen and progesterone. Representation of the mean ± standard error of the mean corresponding to the densitometric values of active MMP-2 in ovariectomized mice (Ovx, n = 6), ovariectomized mice treated with E (Ovx+E, n = 4), ovariectomized mice treated with P (Ovx+P, n = 3) and ovariectomized mice treated with estrogen and progesterone (Ovx+E+P, n = 3). In all cases, values were normalized to controls that were considered equal to 1. The increase in the levels of active MMP-2 were statistically significant in Ovx+E+P compared with Ovx (*P *≤ 0.001), Ovx+E (*P *≤ 0.05) and Ovx+P (**P ≤ 0.01). **(d) **Zymogram and densitometric analysis of mean ± standard error of the mean showing activation of MMP-2 by progesterone in ovariectomized PR-A mice (Ovx+P, n = 2) compared with ovariectomized mice (Ovx, n = 2). The increase in active MMP-2 activity was statistically significant (*P ≤ 0.05). AMMP, active matrix metalloproteinase; LMMP, latent matrix metalloproteinase; *n*, number of glands analyzed from different mice.

Remodeling of the ECM is regulated mainly by matrix metalloproteinases (MMPs), the expression of which is under developmental regulation in the mammary gland [19]. Furthermore, targeted overexpression of MMP-3 (stromelysin-1) in mammary epithelium leads to increased branching morphogenesis [20], hyperplasia [21] and tumors [22]. To assess whether the loss in BM integrity observed in the mammary glands of PR-A transgenics was due to altered expression of MMPs, we examined the latent and active forms of MMPs by gelatin zymography. As shown in Figure 6a, total tissue extracts of mammary glands derived from either wild-type and PR-A mice revealed three distinct bands corresponding to latent MMP-9, latent MMP-2 and active MMP-2. Quantitation of the levels of total MMP-2 (Figure 6a) and MMP-9 (data not shown) as determined by densitometry did not reveal a significant difference between the mammary glands of wild-type mice and PR-A transgenics. To further investigate whether there could be a link between the total levels of PR and MMP activity, we analyzed tissue extracts derived from PR null mutant mice and compared them with their littermate controls. As shown in Figure 6b, there was a statistically significant decrease in the levels of total MMP-2 in the PR null mutant mice - suggesting that the steady-state levels of PR modulate proteolytic activity in the mouse mammary gland.

To determine whether signaling through ER and/or PR were involved in modulating MMP activities, we analyzed tissue extracts of ovariectomized mice exposed to exogenous estrogen and/or progesterone (Figure 6c). Mammary glands of wild-type mice treated with estrogen and progesterone exhibited statistically significant increased levels of active MMP-2 (Figure 6c). This increase did not occur in the glands derived from animals treated with either estrogen or progesterone alone. As with wild-type mice, MMP-2 activation was observed in mammary glands of ovariectomized PR-A mice treated with estrogen and progesterone; again estrogen alone did not have any effect on the levels of active MMP-2 (data not shown). Progesterone alone, however, elicited MMP-2 activation in PR-A transgenics (Figure 6d).

**Figure 7:**
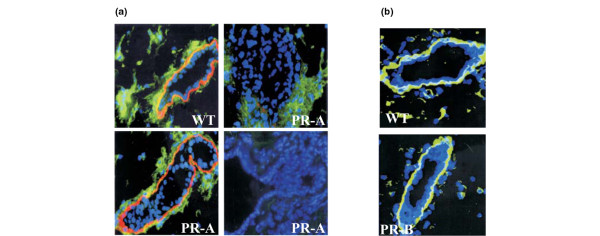
MMP-2/laminin-5 in mammary glands of adult wild-type and PR-A mice; laminin immunostaining in PR-B transgenics.  *In situ *localization of MMP-2 and laminin-5 in mammary glands of adult wild-type (WT) and PR-A transgenics, and laminin staining in PR-B transgenics. **(a) **MMP-2 and laminin-5 were detected in fixed frozen sections of mammary glands, using an indirect immunofluorescence assay, as described in the text. Blue, 4',6-diamino-2-phenylindole (DAPI) staining of the nuclei; green, fluorescein isothiocyanate staining corresponding to immunoreactive latent MMP-2; red, Texas Red staining corresponding to immunoreactive laminin-5. Staining in glands from WT mice and in normal structures derived from PR-A mice showed continuous laminin-5 staining and the presence of MMP-2 in the periepithelial stroma. In aberrant structures derived from glands of PR-A transgenic mice, however, staining for both laminin-5 and latent MMP-2 was lost (right panels). Top left, mammary duct from wild-type mouse; bottom left, normal duct from PR-A transgenic mouse; top right, duct from PR-A transgenic mouse containing both normal and morphogenetically disturbed epithelium; bottom left, aberrant structure in PR-A transgenic mouse. **(b) **Laminin staining (green) in a mammary gland derived from a PR-B transgenic mouse shows that it circumscribes the epithelium as in the WT control. Nuclei were counterstained with DAPI (blue).

To detect MMP activity within the tissue it is necessary to use a tool that can distinguish between the latent and active forms. We used an antibody that specifically detected latent MMP-2 and does not cross-react with its active form. We correlated the localization of latent MMP-2 to that of laminin-5 staining, which has been previously shown to be degraded by MMP-2 [23]. In mammary glands of wild-type mice, and in regions where the ducts appeared normal in PR-A transgenics, latent MMP-2 and laminin-5 were detected in the periepithelial stroma (Figure 7a). In regions with aberrant structures, however, latent MMP-2 was either reduced or dramatically absent and laminin-5 was essentially degraded. These observations that hyperplasias in the mammary glands of PR-A transgenics have increased MMP activity and increased BM degradation support the hypothesis that PR-A drives MMP-2 activation.

Finally, to exclude the possibility that the degradation of the BM was specifically due to overexpression of PR-A and was not the result of the overall imbalance in the ratio of the two isoforms of PR, we carried out laminin-1 staining in mammary glands of PR-B mice. As shown in Figure 7b, the basement membrane was intact in PR-B mice.

## Discussion

We have previously shown that mammary gland development in PR-A transgenic mice is abnormal, characterized by extensive ductal growth, lateral branching and loss of basement membrane integrity and cell-cell adhesion [6]. In the present report we show that the proliferative phenotype of PR-A mice is dependent on ovarian steroids, where hyperplasia is mediated by distinct mechanisms. PR-A transgenic expression augments ER-α mediated proliferation while PR-A the degradation of the basement membrane, both essential requirements for the development of hyperplasias. Moreover, even though progesterone is required for the manifestation of the hyperplasias, antiprogestins have no effect on the established hyperplastic phenotype.

Signaling through estradiol/ER is required for proliferation, but is not enough to give rise to overt hyperplasias. Once established, these hyperplasias are dependent on ER signaling, since treatment with the antiestrogen ICI 182,780 abolishes them; we have not determined, however, the relative role of ERα and ERβ. On the other hand, the overexpression of PR-A leads to antiprogestin resistance, since neither ZK 98,299 nor RU486 had any effect on the expression of the hyperplasia in the intact mice. Both antiprogestins have similar mechanisms of action inducing PR binding to the DNA, but causing conformational changes that block co-activator binding to the receptor, thus making it transcriptionally inactive [24]. The antagonistic activity in both cases, however, has been shown to depend on the cellular context that determines the degree of the antagonist/agonist activity of these compounds, which relies on the balance between expression and availability of coactivators and corepressors and the context of specific target promoters available in any given cell type [24]. Furthermore it is clear from the ovariectomized mice that progesterone is necessary to induce the full phenotype. Overexpression of PR-A in the mouse mammary gland therefore seems to generate a cellular context that promotes hyperplasia, but whose maintenance is not dependent on progesterone signaling *per se*.

The hyperproliferation observed in the mammary glands of PR-A transgenic mice may be due to the diminished expression found for both latent and active TGFβ1. Activation of TGFβ1 is regulated by estradiol and progesterone, which in turn restricts the proliferative response to these steroids [14]. Our studies suggest that physiological estradiol drives inhibition of TGFβ1 that in turn increases proliferation in response to estradiol, which is consistent with its action in estrus shown using TGFβ1 heterozygote mice [17]. Notably, the mammary glands of *Tgfb1 *heterozygote mice are two to four times more proliferative yet there is no evidence of hyperplasia [17]. While similar to *Tgfb1 *with regards to decreased TGFβ1 levels and increased proliferative response to steroid hormones, an additional action of PR-A must therefore be necessary for the expression of hyperplasia.

Since our studies show that signaling through progesterone has no impact on proliferation in hyperplastic PR-A mice but is required for the manifestation of the hyperplasias, we postulated that another effect must be required. We show that signaling through PR modulates the activation of MMP-2, and that areas of BM disruption coincide with loss of latent MMP-2. The integrity of BMs is dictated by the composition of MMPs, their inhibitors and the status of their activation [25]. MMPs have been shown to be key regulators of branching morphogenesis in the mouse mammary gland [20,26,27]. On the other hand it has long been known that both estrogen and progesterone affect mammary gland development regulating ductal elongation, side branching and differentiation. The development of PR and ER transgenic and knockout mice in the past decade has provided additional evidence to confirm these results [4,6,28]. To date, however, there is insufficient evidence to address the intriguing question of how hormonal action, MMP production and activation are linked in the mouse mammary gland.

We have previously shown that there was loss of laminin-1 in areas of aberrant epithelial morphology in PR-A transgenic mice [6]. We now report that, in addition to laminin-1, immunoreactivity of laminin-5, collagen IV and collagen III is also lost. In the mammary glands of PR-A transgenics, therefore, the concurrent loss of laminin and collagen indicate that the ECM is broadly affected. More importantly the fact that the phenotype was reversible by ovariectomy points to a role for hormonal action in the maintenance of ECM integrity, which is supported by our observation that the administration of estradiol and progesterone to ovariectomized PR-A transgenic mice led to BM degradation, strongly supporting this notion.

Alterations in the mammary gland ECM have been previously described in transgenic mice expressing an active form of MMP-3 in the epithelium [21]. The fact that total MMP-2 levels in extracts derived from PR-A transgenic mice were not significantly different could be due to the heterogeneous phenotype, as previously shown [6], and is consistent with the statistically significant decrease we observed in MMP-2 levels only in PR null mutant mice. Moreover, immunolocalization studies support the notion of a restricted phenotype as we found decreased latent MMP-2 only in hyperplastic regions where laminin-5 was absent.

The finding that treatment of ovariectomized wild-type mice with estradiol and progesterone leads to the activation of MMP-2 is the first direct demonstration that hormonal action modulates MMP activity in the mouse mammary gland. This result suggests an important role for MMP-2 during proliferation and differentiation that takes place during pregnancy. It is significant to note that while in wild-type ovariectomized mice progesterone alone had no effect on MMP-2 activation (because there is little or no PR-A), it caused an increase in the activation of MMP-2 in mammary glands of ovariectomized PR-A transgenics because they still expressed a high level of PR-A. In wild-type mice, ER would therefore be necessary to support the levels of PR, but signaling through PR-A would be important for the activation of MMP-2.

Using MMP-2 knockout mice, Wiseman and collaborators have shown that during puberty MMP-2 positively regulates invasion of terminal end buds but that it inhibits side branching from mature ducts. They also postulated TGFβ1 as the candidate for the inhibitory action of MMP-2 on lateral branching at puberty [26]. This speculation is supported by recent modeling studies that demonstrate the effect of TGFβ1 as a branching inhibitor [29]. In mammary glands of PR-A transgenics there is a positive relationship between the steroid hormone-dependent increase in MMP-2 activity and lateral branching, accompanied by a reduction in both the latent and active forms of TGFβ1. It is possible that other proteases are also involved, however, given that, although statistically significant, the degree of activation of MMP-2 was modest both in the wild-type and in the PR-A transgenics.

Given that MMP-2 is expressed by the periductal stroma and weakly by the adipose tissue [26], and that PR is expressed exclusively in the epithelial compartment [30], cross-talk between both compartments seems to be a necessary condition for MMP-2 activation. A possible mediator of this cross-talk may be MT1-MMP, which is expressed both by the stroma and the epithelium [26]. This hypothesis is supported by the fact that we have detected an increase in the levels of MT1-MMP in the mammary glands of pregnant mice, and that MT1-MMP levels were high in certain areas in mammary glands of PR-A transgenic mice (Simian M and Shyamala G, unpublished observations). Additionally, degradation of collagen IV appears to require the action of MT1-MMP [31]. Whether PR-positive cells are also MT1-MMP-positive remains to be determined.

## Conclusions

An imbalance in the relative ratio of PR-A and PR-B isoforms has been observed in certain human mammary tumors, and this is often associated with overexpression of the PR-A form [32-34]. Our studies show that this imbalance has a direct impact on three major phenomena underlying mammary biology - that is, steroid hormone responsiveness, TGFβ1 production, and ECM remodeling mediated by MMP activity - all of which are implicated in mammary epithelial cell transformation.

## Abbreviations

BM: basement membrane; BrdU: 5-bromo-2-deoxyuridine; ECM: extracellular matrix; ER: estrogen receptor; FCS: fetal calf serum; H&E: hematoxylin and eosin; LAP: latency-associated peptide; MMP: matrix metalloproteinase; PBS: phosphate-buffered saline; PR: progesterone receptor; PR-A transgenics: mice carrying additional A form of PR; PR-B transgenics: mice carrying additional B form of PR; TGFβ1: transforming growth factor beta 1.

## Competing interests

The authors declare that they have no competing interests.

## Authors' contributions

MS performed the zymography studies, prepared the results and contributed to the drafting of the manuscript. MJB participated in the conception of the study, the direction of the research and the correction of the manuscript. MHB-H participated in the conception of the study, carried out the TGFβ1 studies and the immunofluorescence of the BM components, and corrected the manuscript. GS conceived the study, directed the research, analyzed the data, kept the transgenic mice, carried out the whole mounts and BrdU studies, and drafted and corrected the manuscript.
